# Comorbidity of Glucose-6-Phosphate Dehydrogenase Deficiency and Sickle Cell Disease Exert Significant Effect on RBC Indices

**DOI:** 10.1155/2019/3179173

**Published:** 2019-03-19

**Authors:** Samuel Antwi-Baffour, Jonathan Kofi Adjei, Peter Owadee Forson, Stephen Akakpo, Ransford Kyeremeh, Mahmood Abdulai Seidu

**Affiliations:** ^1^Department of Medical Laboratory Sciences, School of Biomedical and Allied Health Sciences, College of Health Sciences, University of Ghana, P. O. Box KB 143, Korle-Bu, Accra, Ghana; ^2^Department of Medical Laboratory Sciences, School of Allied Health Sciences, Narh-Bita College, Tema, Ghana

## Abstract

**Background:**

Glucose-6-phosphate dehydrogenase (G6PD) converts glucose-6-phosphate into 6-phosphogluconate in the pentose phosphate pathway and protects red blood cells (RBCs) from oxidative damage. Their deficiency therefore makes RBCs prone to haemolysis. Sickle cell disease (SCD) on the other hand is a hereditary blood disorder in which there is a single nucleotide substitution in the codon for amino acid 6 substituting glutamic acid with valine. SCD patients are prone to haemolysis due to the shape of their red blood cells and if they are deficient in G6PD, the haemolysis may escalate. Reported studies have indicated variations in the prevalence of G6PD deficiency in SCD patients and as such further work is required. The aim of this study was therefore to estimate the incidence of G-6-PD deficiency among SCD patients and to determine its impact on their RBC parameters as a measure of incidence of anaemia.

**Methods:**

A total of 120 clinically diagnosed SCD patients of genotypes HbSS and HbSC were recruited into the study. About 5ml of blood was collected via venipuncture from each patient and used to run G6PD, full blood count, and haemoglobin (Hb) electrophoresis tests. The data were analyzed using SPSS version 20 and Graphpad prism.

**Result:**

G6PD deficiency was detected in 43 (35.83%) of the participants made up of 16 (13.33%) males and 27 (22.50%) females of whom 17 (14.17%) had partial deficiency and 10 (8.33%) full deficiency. Statiscally significant differences p=0.036 and p=0.038 were established between the Hb concentration of the participants having a G6PD deficiency and those with normal G6PD activity for males and females, respectively.

**Conclusion:**

From the results obtained, it implies that G6PD deficiency may increase the severity of anaemia in SCD patients. There is therefore the need to screen all SCD patients for G6PD deficiency to ensure that their condition is not exacerbated during treatment.

## 1. Introduction

Glucose-6-phosphate dehydrogenase (G6PD) which is the rate limiting enzyme in the pentose phosphate pathway converts glucose-6-phosphate into 6-phosphogluconate [1]. It protects red blood cells (RBCs) from oxidative damage by supplying reducing energy to them by maintaining the level of reduced co-enzyme nicotinamide adenine dinucleotide phosphate (NADPH) [2]. The NADPH in turn maintains the supply of reduced glutathione (GSH) in the RBCs which acts like oxidant scavenger that is used to mop up any oxidants (free radicals) that will cause damage to the RBCs [3].

G6PD deficiency is an X-linked recessive genetic disorder that occurs most often in males in heterozygous condition and in females in only homozygous condition but can be partial in a heterozygous female [4]. In affected individuals, the defect in the enzyme causes RBCs to break down prematurely, resulting in medical problems such as haemolytic anaemia, which eventually leads to paleness, jaundice, dark urine, fatigue, shortness of breath, and a rapid heart rate [5]. The haemolytic anaemia is mostly triggered by bacterial or viral infections or by certain antimalarial drugs but can also occur after eating fava beans or inhaling pollen from fava plants or fumes from moth balls [6].

Sickle cell disease (SCD) is also a hereditary blood disorder or an autosomal recessive disease caused by a point mutation in haemoglobin by the substitution of valine for glutamic acid at position 6 of the beta (*β*)–globin chain found on chromosome 11 [7]. This makes the red blood cells to assume an abnormal, rigid, sickle-like shape under low oxygen tension [7]. Sickle cell disease or sickle cell anaemia (SCA) as it is sometimes called occurs in an individual when the individual inherits two abnormal copies of the haemoglobin (Hb) genes, one from each parent. Several subtypes exist depending on the exact mutation in the haemoglobin gene. Some occur with a single abnormal copy and the individual does not experience any symptoms and are said to have sickle cell trait or are carriers [8].

In tropical areas, where malaria is endemic, there is high prevalence of haemoglobinopathies including SCD and G6PD deficiency. This prevalence is thought to be related to the selective advantages against malaria of both SCD and G6PD gene as they provide some sort of immunity against malaria parasites especially,* plasmodium falciparum *[9]. Over the years, reported studies have shown that there was a variation of the prevalence of G6PD deficiency in sickle cell disease or sickle cell anaemic patients. Some authors say that the incidence of G6PD deficiency is higher in patients with SCD than the general population without the haemoglobinopathy [10, 11]. Other authors, however, do not agree with this association, believing rather that the mutated genes responsible for both the G6PD and the sickle cell disease do not stay on the same chromosome, so they cannot be linked together [12, 13].

So, the possible relationship between G6PD deficiency and haemoglobin S genotypes among various populations and in individual cases has been a subject of interest in scientific research. Even, back in the 1970s, Piomelli* et al*. (1972) gathered evidence in favor of an association between homozygous sickle cell disease and G6PD deficiency in an American community of African origin [14]. Still, the potential influence of G6PD deficiency upon the manifestation features of patients with SCD is a matter of contention and very problematic as they both cause anaemia. Again, it is suspected that G6PD deficiency in patients with heterozygous haemoglobinopathies such as sickle cell trait can cause it to be expressed like symptomatic sickle cell disease [13, 15]. This, notwithstanding, information on the prevalence rate of G6PD deficiency in sickle cell disease patients in Ghana is not complete. Therefore, this study highlights the incidence of G6PD deficiency in SCD patients and its possible effect on their RBC indices. We believe that the outcome will help guide physicians in the treatment regimen given to SCD patients.

## 2. Methodology

### 2.1. Ethics Approval and Consent to Participate

Ethical approval was obtained from the Ethics and Protocol Review Committee of the School of Biomedical and Allied Health Sciences, College of Health Sciences, University of Ghana. The study was also approved by the Head of Sickle Cell Clinic of the Korle-bu Teaching Hospital before sampling commenced. The purpose of the study was thoroughly explained to participants and only those who gave their informed written consent were recruited. Consent was also obtained from parents/guardians of minors before they were recruited. The participants were informed that their information will be kept confidential and they had the right to withdraw their consent and discontinue participation in the study at any time.

### 2.2. Data Collection, Transportation, and Storage

Five milliliters (5ml) of blood was collected via venipuncture into EDTA anticoagulant tubes. The samples were transported in an ice chest to the haematology laboratory for processing and analysis. Samples not processed straight away were kept in the laboratory refrigerator at 4°C until analyzed within the allowable time (0 – 48hrs).

### 2.3. Procedures

#### 2.3.1. Cellulose Acetate Membrane Electrophoresis

*Principle*. At alkaline pH, haemoglobin is a negatively charged protein and when subjected to electrophoresis will migrate toward the anode (+). Structural variants that have a change in the charge on the surface of the molecule at alkaline pH will separate from HbA. Hemoglobin variants that have an amino acid substitution that is internally sited may not separate, and those that have amino acid substitution that has no effect on overall charge will not separate by electrophoresis. By their separation, they appear as bands representing the constituent allele [16].

*Procedure*. Haemolysate (Hb solution) was prepared from all the patient samples. With the power supply disconnected, the electrophoresis tank was prepared by placing equal amount of tri EDTA Borate buffer in each buffer compartment. Two chamber wicks in the buffer were made wet and one was placed along each bridge support, ensuring that they make good contact with the buffer. The cellulose acetate membrane was soaked by lowering it slowly into a reservoir of the buffer and left for at least 5 minutes. The applicator tips were cleaned immediately before use. The cellulose acetate membrane was taken from the buffer and blotted between two layers of cleaned blotting paper. The cleaned tips applicator was loaded by depressing the tip into the same well twice and applying it first onto some clean blotting paper. The applicator was reloaded with the haemolysate and was applied to the cellulose acetate membrane alongside a control. The cellulose acetate membrane was placed across the bridges. The power supply was connected and electrophoresis was carried out at 350 volts for 45 minutes. The cellulose acetate membrane was stained with ponceaus S for few minutes and was distained with 5% acetic acid. Hb genotypes of the participants were identified and recorded.

#### 2.3.2. G-6-PD Test (Methemoglobin Reduction Method)

*Principle*. The G6PD test is based on the principle that the methemoglobin formed by action of sodium nitrite on red blood cells is reduced back to haemoglobin by the activity of G6PD enzymes and the action of the redox dye, methylene blue on the red blood cells, which activates the pentose phosphate pathway. Incubation of the sample with methylene blue therefore allows stimulation of the pentose phosphate pathway in the subjects with normal G6PD level. In G6PD deficient subject, the block in the pentose phosphate pathway prevents this reduction. The rate of reduction of methemoglobin to oxyhemoglobin is proportional to the G6PD activity [17].

*Procedure*. Three test tubes were used for each individual test and were labeled T for test, P for positive control, and N for negative control. 1ml of blood plus 50ul of sodium nitrite plus 50ul of methylene blue was placed in the tube labeled T. 1ml of blood plus 50ul of sodium nitrite was placed in the tube labeled P. Finally, 1ml of blood plus 50ul of methylene blue was placed in the tube labeled N. The test tubes were incubated at 37°C for 3 hours with hourly mixing interval. After the incubation period, three test tubes were arranged and labelled as before. 0.1ml of physiological saline was dispensed into each tube and 0.1ml of the respective incubated samples was transferred into the tubes accordingly and colour comparison was done [17]. The test results were recorded as G6PD normal when the colour of the test solution was similar to the red colour of the negative control. G6PD full defect was recorded when the colour of the test solution was similar to the brown colour of the positive control and as partial G6PD defect when the colour was midway between normal G6PD activity and G6PD full defect.

#### 2.3.3. RBC Indices Estimation

*Principle*. Blood samples were thoroughly aerated and Complete or Full Blood Count was carried out using the Sysmex Automated Haematology Analyzer (Sysmex Co, Japan) on samples in the EDTA tube, according to the manufacturer's instructions that automatically generated values for WBCs, haemoglobin, lymphocytes, and platelets counts.

### 2.4. Data and Statistical Analysis

Data was collected using notebooks and transferred to a computer and kept confidential. They were later entered into Microsoft Word and analyzed using Statistical Package for Social Sciences (SPSS, Version 20.0) and Graphpad Prism. Normally distributed data were analyzed using independent sample t-test and expressed as mean ± SD. A one-way ANOVA test was used to compare the differences in RBC indices and the different G6PD statuses. Statistical significance was taken as p≤ 0.05.

## 3. Results

**Figure 1:**
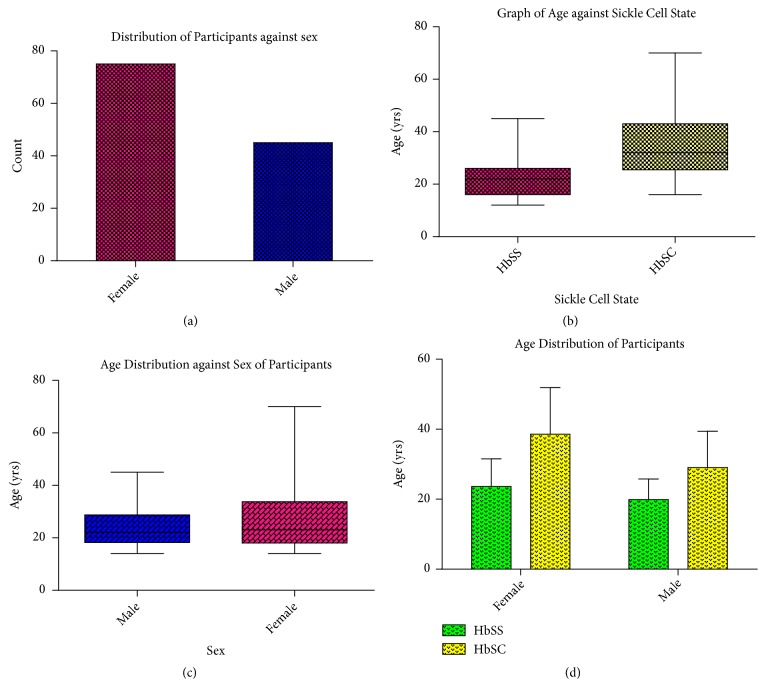
The figure shows the age and gender distribution of the participants. Majority (62.5%) of the participants were females.

A total number of 120 clinically diagnosed sickle cell disease participants were recruited and screened for G6PD deficiency. Eighty (m=27; f=53) participants had the sickle cell disease with genotype Hb SS and the remaining forty (m=18; f=22) had sickle cell disease with genotype Hb SC. Their age range was from 13 to 69 years (Figure 1).

**Figure 2:**
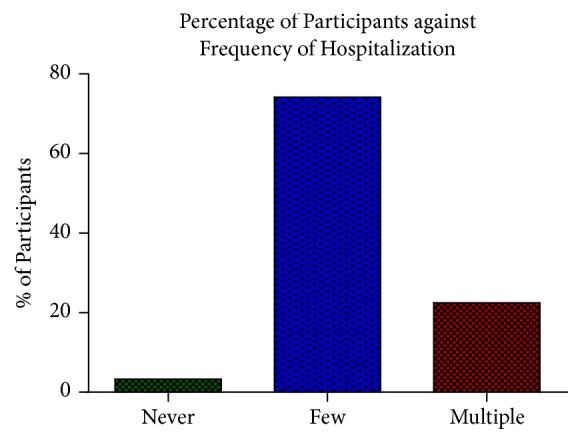
The graph above shows the distribution of the extent to which participants are hospitalized. One (1) represents those who have not being hospitalized, (2) represent those hospitalized for few times, and (3) represent those hospitalized multiple times.

The frequency of hospitalization was assessed and most of the participants said that they were not frequently admitted to the hospital and if even admitted, they spent just a few days. The distribution was as follows: eighty-nine (89) (74.17%) had few times (1-3 times a year) of hospitalization, 27 (22.5%) had multiple (4 – 10 times a year) hospitalization, and 4 (3.33%) said they were new patients and yet to undergo any admission (Figure 2).

Furthermore, out of the total number of the participants, 99 (84.6%) complained of painful crises, 93 (79.56%) complained of headache, 39 (33.3%) complained of shortness of breath, 74 (63.2%) complained of fatigue, and 61 (52.1) complained of dizziness. Others also suffered from other medical problems such as hypertension and asthma as well as frequent anaemia (mainly the SS). Again, one hundred and seventeen (117) (97.5%) of the participants were taking folic acid medication, whilst 3 representing 2.5 % were not taking any medication (Table 1).

**Figure 3:**
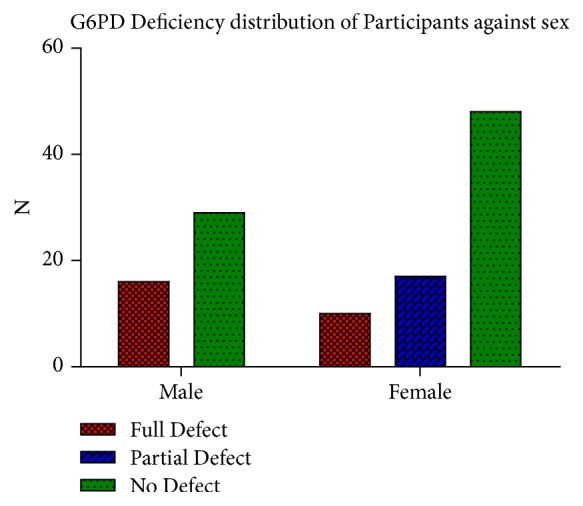
The figure represents G6PD deficiency (full and partial) and G6PD normal among the participants.

The percentage G6PD deficiency among the participants based on their gender was also determined. Twenty-nine (29) (24.17%) of the males and 48 (40%) of the females had no defect, 16 (13.33%) of the males, and 10 (8.33%) of the females had full defect. Then 17 (14.17%) of the females only had partial defect (Figure 3).

Furthermore, G-6-PD defect among the different genotypes was also determined and although the participants with Hb SS were twice as much as those with Hb SC, it turned out that Hb SC genotype had the highest defect with 19 full and 6 partial defects, whilst Hb SS had 14 full and 4 partial defects (Table 2).

Again, an independent sample t-test was conducted to determine the association between the Hb genotype and the mean G6PD defect (both full and partial) and it was found to be insignificant with a p-value of 0.061 (Table 3).

For the haemoglobin (Hb) estimation as indicator for anaemia, Hb concentration between 13.5 and 17.5g/dl was used as the normal for males, whilst 11.5 to 15.5g/dl was used as the normal for females [18]. A concentration above 9.9g/dl but lower than the minimum reference range in both males and females was designated as low level and represented mild anaemia. Then between 7.0 and 9.9g/dl was described as lower level indicating moderate anaemia and one below 7.0g/dl taken as lowest level indicating severe anaemia. Out of the total number of participants, 7 (5.8%) had normal Hb concentration; 21 (17.5%) had low Hb concentration, mild anaemia; 80 (66.7%) had lower Hb concentration, moderate anaemia; and 12 (10%) had lowest Hb concentration, severe anaemia (Table 4).

**Figure 4:**
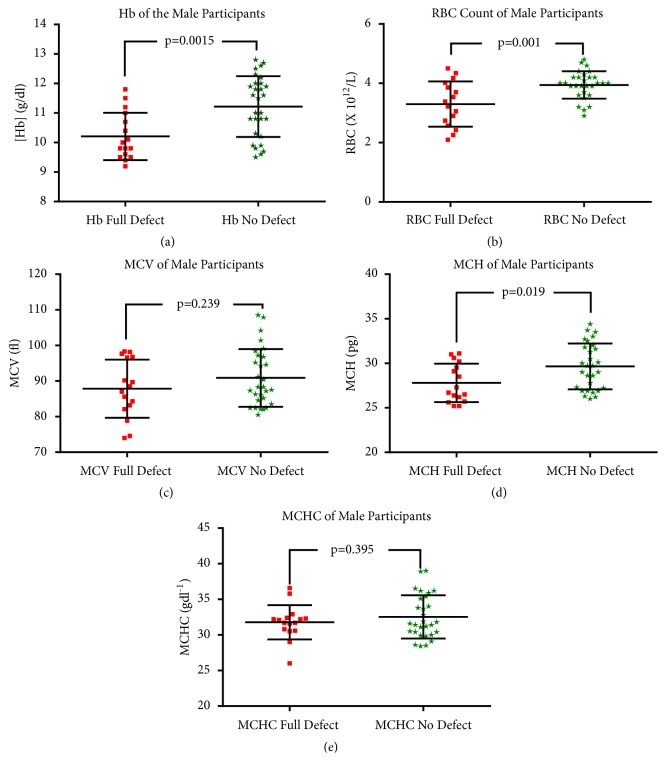
The figure represents comparison of RBC indices and G6PD status of male participants.

**Figure 5:**
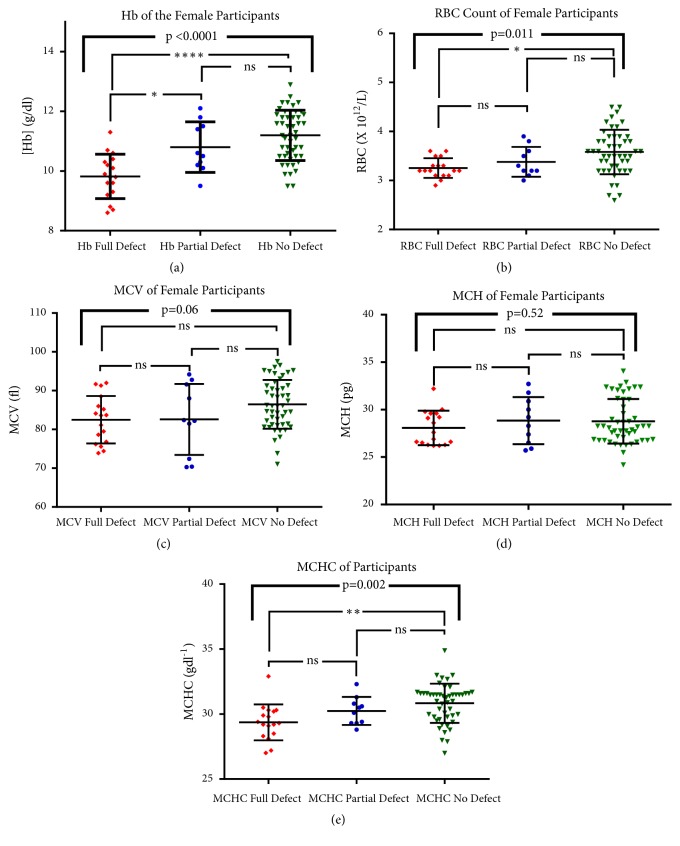
The figure represents comparison of RBC indices and G6PD status of female participants.

From the FBC analysis, the values of RBC indices including Hb were compared between the participants who had G-6-PD full defect, partial defect, and no defect (Figures 4 and 5). Five graphs were generated for each figure representing the mean values of the different RBC indices.

From Figure 4, the results indicate that the mean Hb showed significant difference between the participants with full defect and those with no defect, p=0.0015 (graph (a)). From graph (b), the mean RBC was also significantly different between the participants with full defect and those with no defect (p=0.001). From graph (c), no significant difference was seen between the males with full defect and those without the defect when MCV was evaluated (P=0.239). Graph (d) evaluated MCH and here significant difference was recorded (p=0.019). Finally, from graph (e), the MCHC showed significant difference between males with full defect and those with no defect (p=0.395).

From Figure 5, graph (a) showed significant difference in mean Hb between the participants with full defect and those with no defect (p=<0.0001). However, no significant difference was seen between participants with partial defect and those with no defect as well as full defect and partial defect participants. From graph (b), the mean RBC also showed significant different between the participants with full defect and those with no defect (p=0.011), but no significant difference between full defect and no defect as well as full defect and partial defect. From graphs (c) and (d), no significant difference was seen between the participants with full defect, partial defect, and no defect when MCV and MCH were evaluated, respectively (p=0.06 and 0.52). Graph (e) on the other hand shows significant difference in MCHC between full defect and partial defect (p=0.002) but no significant difference between full defect and no defect as well as partial defect and no defect.

## 4. Discussion

The current study examined 120 individuals that have been clinically diagnosed with sickle cell disease (SCD). Forty-five (37.50%) out of the total population were males and seventy-five (62.50%) were females (Figure 1). Majority of the participants are hospitalized 1–3 times a year, whilst others are hospitalized 4–10 times a year and some are yet to be hospitalized (Figure 2). All the participants had one or more complaints that compounded their condition (Table 1). Also, 80 (66.7%) were SCD patients with Hb SS whilst 40 (33.3%) of them had Hb SC. G6PD full defect was detected in 16 (13.33%) males and 10 (8.33%) females and partial defect was detected in 17 (14.17%) of the females. This implies that, together, G6PD deficiency was detected in 43 (35.83%) of the participants (Figure 3). Again, although majority of the participants were of the SCD genotype SS, most of the G6PD deficiencies were found in the participants with genotype SC with a mean deficiency of 1.78 ± 0.89 (Tables 2 and 3)

The prevalence obtained in this study is similar to those obtained by other studies done by different authors in different countries on SCD patients. For example, a study detected G6PD deficiency in 20 (27.03%) out of 74 patients with major sickle cell disease (MSCS) and 26 (18.57%) out of 114 individuals with sickle cell trait (SCT) in Burkina Faso [13]. According to Chan (1996), G6PD deficiency detected in males with SCD in Nigeria was 2–24%, also 2-25% among males in Kenya, and about 24% in Ghanaian males [19]. In Ibadan, in Nigeria, G6PD deficiency was detectd in 16 males out of 100 male participants with SCD, also in 25%, 24%, and 19% among 56, 54, and 24 sickle cell anemia patients in Chicago, New York, and Los Angeles, respectively, in USA. Again, 22% out of 41 sickle cell anaemia patients were found to be G6PD-deficient in Lubumbashi, Zaire [20]. On the other hand, our prevalence is lower than that detected in Ghana in 1966, which was 43% among 95 sickle cell anemia patients [21].

In terms of gender, the prevalence of G6PD full deficiency of 35.56% among the male participants was higher than that found in the female participants (13.33%), even though the female participants were more than the males (Figure 3). This finding is similar to that of Jacques et al. (2007) which was 20.5% among males and 12.3% among females with a signifiant p-value of 0.04 [13]. The occurrence of more full defects in males is due to the fact that the males are hemizygous whilst females are dizygous for the X chromosome and, as such, the probability of finding the genes for the G6PD mutation on the two X chromosome is lower [22, 23]. Furthermore, G6PD enzymatic activity between male and female is due to the fact that the phenomenon of lionization activates only one X chromosome in each female's cell [22, 23]. The findings in this study also showed that Hb SC participants had a mean G6PD deficiency of 1.78±0.89 higher than the mean G6PD deficiency of the Hb SS participants (1.46±0.78). There was therefore a slight and insignificant difference of the G6PD deficiency in Hb SS and Hb SC genotypes (p=0.061) (Table 3).

From the FBC analysis, the haemoglobin concentration of Hb SC participants was mostly found in the normal and the low-level ranges and that of Hb SS participants was found in the mildly low and the severely low range (Table 4). This showed the presence of anaemia amongst some of the participants. However, this finding was not surprising since G6PD deficiency and SCD or SCA are two genetic disorders of red blood cells which predisposes an individual to haemolytic anaemia. This finding was in line with the study of Lionnet et al. (2012), which showed that Hb SC individuals have higher haemoglobin concentration than the Hb SS individuals [24].

When all the RBC indices were compared between the male participants according to their G6PD status, it was seen that the mean Hb, RBC, and MCH showed significant difference between the participants with full defect and those with no defect. However, no significant difference was seen between the males with full defect and those without the defect when MCV and MCHC were evaluated. On the part of the females, significant difference was seen in mean Hb, RBC, and MCHC between the participants with full defect and those with no defect. However, no significant difference was seen between the participants with full defect, partial defect, and with no defect when MCV and MCH were evaluated.

According to some writers, G6PD deficiency does not offer any merit or demerit to patients with sickle cell disease or sickle cell anemia [21]. Nevertheless, such conclusion should not lead us to overlook the fact that SCD patients with G6PD deficiency run the significant risk of developing haemolytic crises, sepsis, haemoglobinuria, and kidney failure after taking anti-malarial drugs or eating oxidative foods like fava beans. This, coupled with our findings of decreased RBC indices in comorbid patients, indicate that it may be very important to screen for G6PD deficiency among SCD patients in order to provide them with the appropriate therapeutic and preventive measures.

### 4.1. Conclusion

This study places the prevalence of glucose-6-phosphate dehydrogenase deficiency among sickle cell disease patients at 35.83% which indicate a significant G6PD deficiency among SCD patients. There was also a significant difference between the RBC indices of G6PD normal and G6PD deficient participants. This implies that G6PD deficiency may increase the severity of anaemia in SCD patients. It may therefore be important for SCD patients to know their G6PD status in order to avoid consuming drugs and foods that might provoke oxidative stress in their RBCs.

## Figures and Tables

**Table 1:** A table presenting the clinical data of participants.

Variables	Frequency (n)	Percentage (%)
Painful Crisis	99	84.6
Headaches	93	79.56
Shortness of Breath	39	33.3
Fatigue	74	63.2
Dizziness	61	52.1

Other Complication		
Hypertension	5	4.2
Asthma	4	3.3

Frequent Anaemia		
Yes	22	18.3
No	98	81.7

Folic Acid Treatment		
Yes	117	97.5
No	3	2.5

**Table 2:** G-6-PD defects among the different genotypes.

Hb Genotype	Males (n = 16)	Females (n = 27)	
	Full defect	Full defect	Partial defect
Hb SS	6 (5%)	8 (6.7%)	4 (3.3%)
HB SC	10 (8.3%)	9 (7.5%)	6 (5%)
Total	16 (13.3%)	17 (14.2%)	10 (8.3%)

**Table 3:** The table shows a comparison of G6PD deficiency among the haemoglobin genotypes of the participants.

Hb genotype	Mean G6PD defect ±SD	95% CI	t-test	P-value
Hb SS	1.48 ± 0.78	(1.12, 1.84)	-1.984	0.061
Hb SC	1.78 ± 0.89	(1.43, 2.13)		

*∗P < 0.05 is considered significant.*

**Table 4:** The distribution of haemoglobin concentration of the participants.

Haemoglobin level	Frequency (n)	Percentage (%)
Normal	7	5.8
Low (mild anaemia)	21	17.5
Lower (moderate anaemia)	80	66.7
Lowest (severe anaemia)	12	10

## Data Availability

The datasets used and/or analysed during the current study are available from the corresponding author on reasonable request.
